# Memantine Improves Attentional Processes in Fragile X-Associated Tremor/Ataxia Syndrome: Electrophysiological Evidence from a Randomized Controlled Trial

**DOI:** 10.1038/srep21719

**Published:** 2016-02-22

**Authors:** Jin-Chen Yang, Annette Rodriguez, Ashley Royston, Yu-Qiong Niu, Merve Avar, Ryan Brill, Christa Simon, Jim Grigsby, Randi J. Hagerman, John M. Olichney

**Affiliations:** 1 Center for Mind and Brain, University of California Davis, Davis, CA, 95618 USA; 2 Department of Neurology, University of California Davis, School of Medicine, Sacramento, CA, 95817 USA; 3 Department of Psychology, California State University, Sacramento, Sacramento, CA, 95819 USA; 4 Department of Psychology, University of California Davis, Davis, CA, 95616 USA; 5 University of Vienna, Vienna, 1010 Austria; 6 Department of Psychology, Department of Medicine, University of Colorado Denver, Denver, CO, 80217 USA; 7 Medical Investigation of Neurodevelopmental Disorders (M.I.N.D.) Institute, University of California Davis, School of Medicine, Sacramento, CA, 95817 USA; 8 Department of Pediatrics, University of California Davis, School of Medicine, Sacramento, CA, 95817 USA

## Abstract

Progressive cognitive deficits are common in patients with fragile X-associated tremor/ataxia syndrome (FXTAS), with no targeted treatment yet established. In this substudy of the first randomized controlled trial for FXTAS, we examined the effects of NMDA antagonist memantine on attention and working memory. Data were analyzed for patients (24 in each arm) who completed both the primary memantine trial and two EEG recordings (at baseline and follow-up) using an auditory “oddball” task. Results demonstrated significantly improved attention/working memory performance after one year only for the memantine group. The event-related potential P2 amplitude elicited by non-targets was significantly enhanced in the treated group, indicating memantine-associated improvement in attentional processes at the stimulus identification/discrimination level. P2 amplitude increase was positively correlated with improvement on the behavioral measure of attention/working memory during target detection. Analysis also revealed that memantine treatment normalized the P2 habituation effect at the follow-up visit. These findings indicate that memantine may benefit attentional processes that represent fundamental components of executive function/dysfunction, thought to comprise the core cognitive deficit in FXTAS. The results provide evidence of target engagement of memantine, as well as therapeutically relevant information that could further the development of specific cognitive or disease-modifying therapies for FXTAS.

Prevalence of the fragile X gene (*FMR1*) premutation is ~1:150 in females and ~1:450 in males of the general population^1^. A portion of older *FMR1* premutation carriers (~40% of males, ~16% of females) develop fragile X-associated tremor/ataxia syndrome (FXTAS), with intention tremor, cerebellar ataxia, polyneuropathy, and cognitive deficits—particularly executive dysfunction—as the primary symptoms^2^. Currently, medical intervention for FXTAS is limited to symptom management^2^,^3^ (Hagerman *et al.* 2013).

Pronounced cognitive deficits in executive functioning, processing speed, and working memory are common in FXTAS^4^,^5^. Attention deficits modulated by *FMR1* premutation are frequently self-reported by premutation carriers^6^. Moreover, the premutation has been associated with Autism and ADHD^7^,^8^. Inhibition and working memory are also impaired in carriers asymptomatic of FXTAS^9^,^10^. Radiological and neuropathological studies in FXTAS revealed abnormalities in prefrontal and fronto-parietal regions crucial for top-down attentional control and working memory^11^,^12^,^13^.

Cellular neuropathological studies have demonstrated abnormal neuronal response to glutamate in the *FMR1* premutation^14^,^15^. In human induced pluripotent stem cell (iPSC)-derived neurons carrying the premutation, Liu and colleagues documented an increased response to glutamate, and higher amplitude and more frequent calcium spiking activity^16^.

Memantine, an uncompetitive NMDA glutamate receptor antagonist approved for the treatment of moderate to severe Alzheimer’s disease^17^, has thus been tested in the first randomized, double-blind, placebo-controlled clinical trial conducted in FXTAS. The main analyses showed no significant treatment effects on the primary outcome measures of intention tremor and executive dysfunction^18^. However, in a substudy utilizing cognitive event-related potentials (ERPs), memantine-associated benefits on both cued-recall memory and N400 repetition effect (an electrophysiological marker of semantic priming and verbal memory processes) were demonstrated^19^, indicating that ERPs offered a more sensitive measure of changes in cognitive processes compared to standard behavioral and neuropsychological tests. This notion is consistent with the suggestions that ERPs can provide a non-invasive and reliable approach to studying the effects of pharmacological interventions on neural processes^20^,^21^. In the present study, effects of memantine treatment on attentional processes that are fundamental to executive function/dysfunction, the cardinal cognitive deficit in FXTAS, were investigated using ERPs obtained from an auditory “oddball” task, an extensively-studied paradigm engaging attentional processes^22^,^23^. In the auditory oddball paradigm utilized in the current experiment, patients were instructed to detect the infrequent “oddball” tone embedded in a train of non-target standard tones. Subjects were instructed to press a button to each target detected and also keep a mental count of the number of targets in that experimental block.

Our prior studies in premutation carriers using the same “oddball” paradigm have demonstrated an altered frontal P300 (P3) ERP component in FXTAS patients^9^,^24^, which tracks their executive dysfunction. The earlier abnormalities of prolonged N100 latency and reduced P200 (P2) amplitude were also found in a predominately male FXTAS group^24^ but not in female premutation carriers asymptomatic of FXTAS^9^. Based on both the NMDA modulation effects on auditory ERPs in animals^25^,^26^,^27^,^28^ and humans^29^,^30^,^31^,^32^, and our prior findings in FXTAS^9^,^24^, we hypothesized that 1-year of memantine treatment would improve attentional abilities as indexed by P2 and P3 measures in the present study.

## Results

Table 1 summarizes the group characteristics at baseline for 24 patients from each group (i.e., memantine and placebo) analyzed in the present study.

### Behavioral performance

The response time (RT) and accuracy data for target detection at two visits and the change over one year (1-year minus baseline) are summarized in Table 2, with no statistically significant differences found for the RT or target detection accuracy between two groups (*p*’s ≥ 0.26). ANOVA of *d*′, the sensitivity for target detection calculated using signal detection theory (*d*′ = normalized hit rate minus false alarm rate^33^), showed an insignificant trend of *treatment* × *visit* interaction (*p* = 0.13), with an increase in the memantine group, but a slight decrease in the placebo group. The marginially enhanced sensitivity for target detection as reflected by the *d*′ suggests a beneficial effect from the memantine treatment.

There was no group difference on the |count-hit| discrepancies (a behavioral attention/working memory measure obtained from the dual-response oddball task, contrasting the total correct button-presses and the reported mental count of targets) at either the baseline or follow-up visit (*p* = 0.79), however, ANOVA revealed a significant *visit* × *treatment* interaction (*F*_1,46_ = 8.64, *p* = 0.005, effect size *η*^2^ = 0.16). Follow-up within-group paired-samples *t*-tests showed that the |count-hit| discrepancy significantly decreased after 1-year treatment for the memantine group (*t*_23_ = 3.0, *p* = 0.006), but not for the placebo group which showed an insignificant increase of the |count-hit| discrepancy (*t*_23_ = −1.32, *p* = 0.20). This result reflects improved attention and/or working memory after treatment with memantine.

### ERP Results

**Figure 1:**
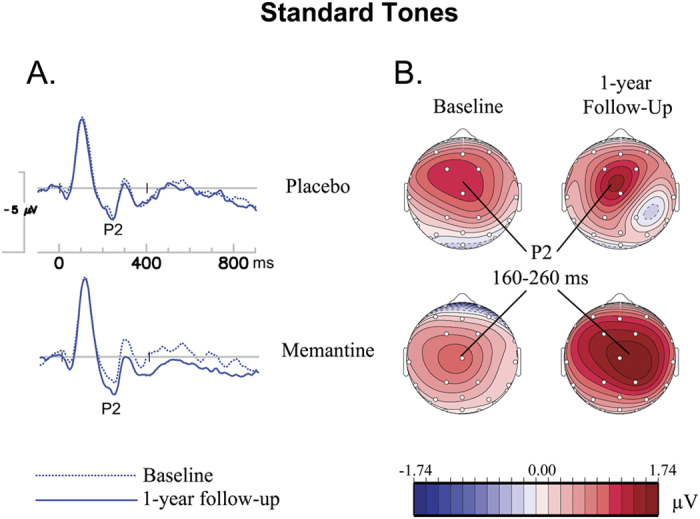
ERPs to standard tones. (**A**) ERPs to standards at the vertex (Cz). (**B**) Topographic maps of P2 amplitude to standard tones, 160–260 ms post-stimulus.

**Figure 2:**
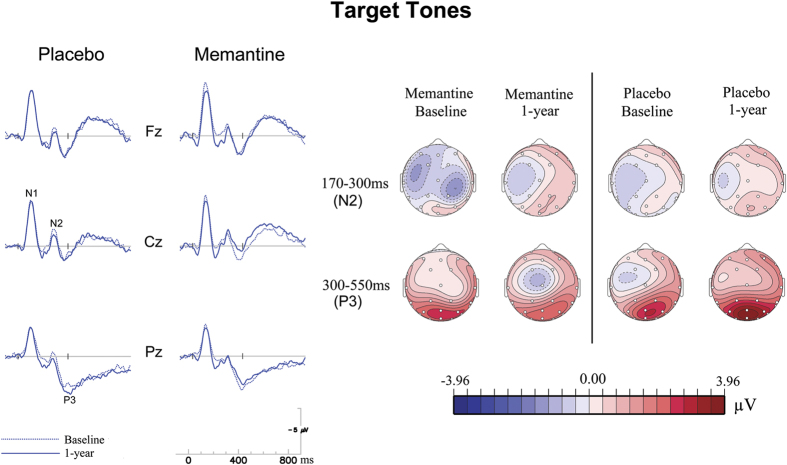
ERPs to targets. ERPs to targets at 3 midline electrodes (left panel), and topographic maps to targets across the N2 (upper) and P3 (lower) time windows (right panel).

Figures 1 and 2 depict the grand average ERPs to standard tones at the vertex (Cz) and the ERPs to target tones at 3 midline electrodes (i.e., Fz, Cz and Pz), respectively. In repeated-measures ANOVAs of the P2 data, a significant *visit* × *treatment* interaction was found for P2 amplitude (*F*_1,44_ = 4.26, *p* = 0.045, *η*^2^ = 0.88). Post-hoc analyses found significantly larger increases of the composite P2 amplitudes (averaged from the fronto-central cluster of 4 electrodes Fz, Cz, and FC1/2) in the memantine group compared to the placebo group (1-year – baseline: memantine group = 0.79 ± 1.3 *μ*V, placebo group = −0.01 ± 1.2 *μ*V; *t*_46_ = 2.19, *p* = 0.03). Post-hoc within-group paired-samples *t*-tests showed that the P2 amplitude was significantly enhanced after 1-year intervention for the memantine group (*t*_23_ = −2.88, *p* = 0.008), but not for the placebo group (*t*_23_ = 0.05, *p* = 0.96). No significant treatment effects were found for other ERP measures analyzed.

**Figure 3:**
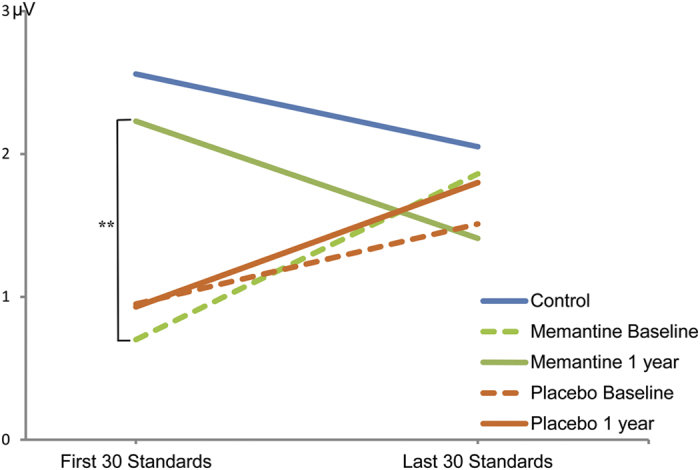
P2 amplitudes (*μV*) to the first 30 standard tones and the last 30 standard tones within the first block of each EEG study, showing a reduced amplitude of responses to the later stimuli (i.e., a habituation effect) in the memantine-treated FXTAS group (solid green) and a normal control group (blue), but increased amplitudes to later stimuli in the other groups. (**p = 0.002).

The ANOVA of P2 habituation effect (i.e., smaller response to later stimuli) found a significant *treatment* × *visit* × *trial position* interaction (*F*_1, 46_ = 4.17, *p* = 0.049). Paired-samples *t*-tests revealed larger P2 amplitude in response to the first 30 standard tones at the 1-year follow-up than at the baseline in the treated group (Fig. 3, mean increase across 4 electrodes: 1-year − baseline = 1.53 *μ*V, *t*_*23*_ = −3.56, *p* = 0.002), but not in the placebo group (mean decrease: 1-year − baseline = −0.02 *μ*V*, t*_*23*_ = −0.63, *p* = 0.54). Furthermore, FXTAS patients who received the memantine treatment exhibited a normalized trend of habituation at the 1-year follow-up (1^st^ 30 tones − last 30 tones = 0.82 *μ*V) comparable to that in a group of 16 age-matched normal controls (0.51 *μ*V). In contrast, FXTAS patients without an active memantine treatment (i.e., the placebo group as well as the treated group at baseline prior to the start of memantine) showed an insignificant trend of a reduced P2 amplitude as well as a smaller and inversed P2 habituation effect compared to normal controls: P2 raw amplitude (averaged across the 4 electrodes) in FXTAS = 0.89 *μ*V, controls = 1.65 *μ*V (*t* = 1.69, *p* = 0.095); P2 habituation effect in FXTAS = −0.82 *μ*V, NC = 0.51 *μ*V; *t* = 1.78, *p* = 0.08), indicating that the increased P2 amplitude and habituation effect in FXTAS after memantine treatment reflect beneficial changes towards normalized brain responses.

### Correlation Results

Correlations between CGG repeat length and the P2 measures were tested using linear regression. Across all patients, CGG repeat length was inversely associated with the composite P2 peak amplitude at the baseline visit (*B* = −0.046, *r* = −0.39, *p* = 0.014), suggesting a link between the FMR1 premutation and P2.

Across all participants, a significant correlation (*B* = −0.178, *r* = −0.32, *p* = 0.025) was observed between the 1-year increase in the composite P2 amplitude (1-year − baseline) and the improvement (decrease from baseline to 1-year) on the behavioral measure of attention/working memory during the oddball task (i.e., the |count-hit| discrepancy). A similar trend of correlation was found within the memantine group (*B* = −0.231, *r* = −0.35, *p* = 0.09), but not in the placebo group (*B* = −0.60, *r* = −0.12, *p* = 0.56).

## Discussion

No targeted treatment has yet been proven effective for FXTAS, a progressive adult-onset neurodegenerative disorder affecting many older *FMR1* premutation carriers. The first report made based upon the larger cohort from which the current study was drawn^18^ concluded that memantine, an NMDA glutamate uncompetitive antagonist, showed no significant effects in FXTAS on the primary clinical and neuropsychological outcome measures of intention tremor severity and executive function. However, herein we have reported that patients with FXTAS administered memantine for one year showed enhanced neural correlates of attentional processes at the stimulus identification/discrimination stage (i.e., the P2 ERP component obtained from an auditory oddball experiment). Increases in P2 amplitude correlated with improved behavioral performance on an attention/working memory measure obtained from the oddball task (i.e., the |count-hit| discrepancy). These improvements were not observed among the placebo group, which exhibited a slight decrement in attention/working memory performance after one year. Thus, we interpret these results as showing that memantine achieved “target engagement”, and the P2 can provide a “surrogate marker”^21^,^34^ for treatment response with improvements in cognitive domains of attention and working memory. However, one should also note that the cognitive effects of memantine were modest, highly significant for count-hit discrepancy, with insignificant trends towards improvement on response time (RT) and target detection accuracy. Additionally, across all patients, the P2 amplitude at baseline was negatively correlated with CGG repeat length. Although we also expected a treatment effect on the P3 measures, the results did not provide support for it. Our prior studies in *FMR1* premutation carriers demonstrated reduced P2 amplitude and an elevated |count-hit| discrepancy in a predominately male FXTAS group^24^, as well as a higher than normal |count-hit| discrepancy in older female *FMR1* premutation carriers without FXTAS^9^. The findings of the present study indicate that 1-year of chronic treatment with memantine might reverse some of the attentional processing deficits in the premutation carriers with FXTAS.

The precise nature of the auditory P2 is not fully understood, but it is generally agreed that the P2 is sensitive to processes beyond sensation and that attention has significant modulatory effects on this component^35^,^36^. P2 sensory gating has been related to brain mechanisms that filter potentially interfering stimuli in attention allocation. Studies using selective attention tasks suggested that the P2 reflects inhibitory control of automatic access to distractors^37^, or allocation of marginal attentional resources needed for non-target stimuli to be identified and rejected^38^,^39^. Enhanced P2 amplitudes after training have been linked to increased distractor inhibition^40^,^41^. Consistent with these prior studies, the memantine-related P2 amplitude increase in the current study likely represents improved distractor inhibition and/or similar processes at an earlier stage of stimulus identification/discrimination. The improved P2-indexed activities subsequently benefited the attentional processing of targets, resulting in treatment-related gains in attention/working memory performance as measured by the reduced |count-hit| discrepancy, the trend of better sensitivity (*d*′) for target detection, and normalized habituation of P2 amplitude. The improvements in these behavioral and neural measures, as well as the positive correlation between changes in P2 amplitude and |count-hit| discrepancy during the oddball task, indicate that the memantine-associated changes obtained in the current study represent beneficial treatment effects for FXTAS. Thus, our present findings support the hypothesis that 1-year of memantine treatment has positive effects on some fundamental components of executive function/dysfunction^42^,^43^, which are thought to comprise the core cognitive deficits in FXTAS^5^.

Unlike most ERP components, P2 amplitude has been found to increase with normal aging, a phenomenon interpreted as an inappropriate orienting response to irrelevant stimuli and a deficient capacity to withdraw attentional resources from irrelevant stimuli^36^,^39^ (but also see Polich^44^ for a lack of age effect on P2). The aging-related amplitude increase might have cancelled the FXTAS-associated decrease^24^ and explains that no P2 amplitude reduction was found for the carriers on placebo for one year.

The primary generators of the auditory P2 have been localized in auditory cortex including the lateral Heschl’s gyrus^36^. It is also thought that the thalamo-midbrain reticular activating system and insula are important for P2 generation^45^,^46^. Notably, radiological studies in FXTAS have found alterations in several putative P2 generators including insula, superior temporal lobe, and reticular activating system structures (e.g., thalamus and cerebral peduncle)^47^,^48^. Thus, it is plausible that the NMDA antagonist memantine ameliorates neuronal abnormalities in one or several of the putative P2 generators that have a high density of NMDA receptors, such as the reticular activating system.

The middle and late-latency cortical ERPs in rodents have shorter latencies but very similar morphology and at least partially shared functional significance when compared to human auditory ERP components^27^. The finding of a specific effect of memantine on the P2 among several auditory ERP components is in line with a number of prior pharmacologic studies primarily in mice, which have implicated glutamate as having a central role in modulations of auditory P2. For instance, early postnatal treatment of mice with NMDA antagonist phencyclidine (PCP) has been shown to significantly disrupt auditory gating of the P2, but not N1 or P1 amplitude^49^. Depending on the specific type and dosage of the NMDA drug administered, P2 amplitude can be either enhanced or attenuated. Tikhonravov and colleagues^25^ have found that low-dose memantine increased the P2 and N2 amplitudes to deviants. The NMDA antagonist ketamine has been shown to reduce the auditory P2 amplitude^50^. NMDA receptor subunit NR1 knock-out mice demonstrated reduced NMDA signaling, as well as a surprising absence of the auditory P2^51^. Thus, along with the recent report showing effects of memantine on cued memory retrieval and the N400 ERP repetition effect^19^, our studies support the hypothesis that memantine might reduce *FMR1* premutation-associated abnormalities in glutamatergic signalling and improve cognition in patients with FXTAS.

In summary, our ERP and behavioral data suggest that memantine treatment has a beneficial effect primarily on cognitive abilities, rather than on the more obvious and characteristic motor abnormalities in FXTAS, although occasional improvements have been noticed on motor symptoms with memantine treatment of some patients with FXTAS. For example, one case study reported that memantine ameliorated FXTAS symptoms including tremor, ataxia, neuropathy, depression, and anxiety when combined with venlafaxine in a 65-year old female^52^. Our ERP results demonstrate evidence of target engagement for cognitive function, and may warrant further investigations into the effects of memantine in larger FXTAS samples. It would also be of interest to test whether the improvement in P2 amplitude associated with memantine treatment persists after washing-out the drug, a finding which would support a neuroprotective effect.

One should note that our findings of subtle beneficial effects on cognition may not be strong enough to justify the clinical use of memantine in patients with FXTAS, especially without independent replication. A limitation of this ERP substudy is that we analysed ERP data from study completers with technically adequate data at both timepoints. A potential selection bias is thus introduced, favoring patients who may have had more benign clinical courses. However, it should be noted that the proportion of patients with adequate longitudinal ERP data was identical in both treatment arms and most of the reasons for not completing had very similar prevalences in both groups. Thus, like Seritan *et al.*^18^, we detected no systematic difference in drop-out rates between groups and note that memantine was well tolerated. It also remains uncertain whether chronic memantine treatment at earlier stages of FXTAS might affect disease onset or progression, or whether extended treatment will show continued improvement of cognitive function, or eventually, quality of life. However, considering the lack of any proven therapy to date, the cognitive effects of memantine in FXTAS, as revealed by our ERP measures, provide important and therapeutically relevant information that could further the development of specific cognitive or disease-modifying therapies for patients with FXTAS and the premutation carriers affected by other disorders such as fragile X-associated primary ovarian insufficiency.

In addition to its effects on NMDA receptors, memantine also binds to dopamine D_2_ receptors^53^. Since atypical motor parkinsonism is very common in FXTAS^2^,^54^, it is plausible that some of the cognitive effects we observed could be attributed to memantine’s dopaminergic agonist properties. For example, a MEG study^55^ has demonstrated that dopamine antagonists interfere with involuntary attention shifting, and both prolong and diminish the P3a, a positive component whose latency window is close to the P2. Studies of memantine in several neuropsychiatric disorders other than dementia have found positive and promising results (e.g., overall improvements in ADHD in a preliminary open-label study and mood-stabilizing effects in bipolar disorder patients not responsive to standard treatments)^56^,^57^. Therefore, the effects of memantine on psychiatric symptoms in FXTAS should be examined more closely. Furthermore, clinical trials testing additive or synergistic effects using memantine in combination with other therapeutic agents is another area which may be worthy of further exploration.

## Patients and Methods

### Participants

Participants included patients with FXTAS enrolled in a comprehensive research study at the M.I.N.D. Institute, University of California Davis, between 2007 and 2012^18^. The study was carried out in accordance with the approved guidelines/protocols. All the protocols were approved by the University of California Davis Institutional Review Board. Informed written consent was obtained from all subjects prior to their participation. All the evaluations were performed at the M.I.N.D Institute in Sacramento, California, except for the ERP experiments conducted at the Center for Mind and Brain in Davis, California. Further details about the primary clinical trial can be seen in the published report^18^.

From the 88 patients who started the allocated intervention (43 on memantine, 45 on placebo)^18^, 76 (38 in each arm) participated in the ERP substudy. Eleven (11) participants performed the baseline ERP experiment but discontinued the intervention trial because of illness or similar reasons (5 on memantine, 6 on placebo; detailed reasons reported in Seritan *et al.*^18^) and therefore did not have longitudinal ERP data. There were 9 additional subjects who did not complete the follow-up ERP studies (5 on memantine, 4 on placebo) because of transportation issues or inability to schedule an ERP session when the lab was available (participants were from all over the United States and each went through intensive testing and travel generally over a 3-day visit). Therefore, 56 patients (28 in each arm) completed the auditory oddball ERP experiment at both visits (i.e., at the baseline visit right before the intervention, and at the follow-up after staying in the trial for one year). In the memantine group, 4 participants were excluded from further analyses because of either unusable EEG data with excessive artifacts (described below, n = 3) or extended gap between two visits (n = 1; interval = 715 days, compared to the group mean of 356 days). In the placebo group, 4 patients were excluded due to unusable EEG data with excessive artifacts (n = 3) or having a deep-brain stimulator implanted (n = 1). Thus, data from 24 patients from each group were analyzed in the present study (Table 1).

### Intervention

Tablets identical in appearance containing either 5 mg of memantine or placebo were provided by Forest Pharmaceuticals. Titration started with 5 mg/day by mouth for one week, and dosage was increased by 5 mg each week, until the full maintenance dose of 10 mg twice daily (i.e., 20 mg/day) was achieved by day 22. Participants were instructed to keep all other medications unchanged for the duration of the study.

### EEG/ERP data collection

EEG during a two-stimulus auditory oddball experiment was recorded in a sound-attenuated, dimly-lit chamber. Lower (113 Hz) and higher (200 Hz) frequency pure tones were presented at 40 dB above individual hearing level in 4 blocks, each containing 100 tones, with a stimulus onset asynchrony jittered from 1.0–1.5 seconds. Prior to each block, subjects were instructed to respond to the infrequent (probability = 25%) “oddball” tones (high or low target tones, counterbalanced across blocks). A dual task was employed in which subjects were instructed to press a button to each target tone, and to also keep a mental count of the number of targets in each block. The mental count of target tones was reported immediately following completion after each block. 32-channel EEG was recorded with a Nicolet-SM-2000 amplifier (band-pass = 0.016–100 Hz, sampled at 250 Hz). (See Olichney *et al.*^58^ for more details of the EEG montage used).

### Data Analysis

The |count-hit| discrepancy in each block (i.e., the absolute value of the difference between correct button-presses and mental count to target tones within a block) was calculated for each participant, as an inverse measure (i.e., a lower value represents better performance) of attention/working memory performance during the oddball task^9^,^24^.

Event-locked EEG segments contaminated with blinks, eye movements, excessive muscle activity, or amplifier blocking were rejected using a semi-automated computer algorithm. Artifact-free EEG segments of 1024 ms (with a 100 ms pre-stimulus baseline period, and 924 ms post-stimulus onset) were averaged by experimental condition to obtain the ERPs.

Based on our prior findings using the same paradigm^9^,^24^, mean amplitude and local peak latency of 4 ERP components were quantified in the following time windows: N100 (N1, 70–150 ms), P2 (160–260 ms), N200 (N2, 170–300 ms), and P3 (300–650 ms). The waveforms to both target and standard tones were used to measure N1. The P2 was measured from ERPs to standard tones. The N2 component was defined from the difference wave (ERPs to targets minus standards). The P3 was measured from both the difference wave and the ERP waveform to targets.

ERP measures were submitted to repeated-measures ANOVAs (SPSS 22, IBM) with the between-subjects factor of *treatment*, and the within-subjects factors of *visit* and *electrode*. Analyses of N1 and P2 included 4 fronto-central electrodes (Fz, Cz, FC1/2). Five central channels (Cz, FC1/2, CP1/2) were used for the N2 analyses. P3 analyses were carried out with 26 scalp electrodes (all except FP1/2). The Greenhouse-Geiser correction was used to adjust for violations of sphericity, where appropriate.

To further characterize the modulatory effects of memantine on the P2 component, a habituation analysis was conducted for P2 amplitude. P2 mean amplitude in response to the first 30 standard tones was compared to the amplitude of response to the last 30 standard tones within the first block of each study, with the between-subjects factor of *treatment*, and the within-subjects factors of *visit, trial position,* and *electrode*. Data from a group of 16 age-matched normal controls (mean age = 59.7 years, each of whom only underwent one ERP recording) was used to demonstrate the normal habituation effect.

Linear regression was used to examine the correlations between changes (1-year follow-up minus baseline) in the |count-hit| discrepancy and in ERP measures for which significant treatment effects were shown. Correlations between local peak amplitudes of P2 (measured after application of a 30 Hz low-pass filter) and CGG repeats were also tested.

## Additional Information

**How to cite this article**: Yang, J.-C. *et al.* Memantine Improves Attentional Processes in Fragile X-Associated Tremor/Ataxia Syndrome: Electrophysiological Evidence from a Randomized Controlled Trial. *Sci. Rep.* **6**, 21719; doi: 10.1038/srep21719 (2016).

## Figures and Tables

**Table 1:** Group characteristics at Baseline: Mean ± SD.

	Memantine (*N* = 24)	Placebo (*N* = 24)	*p*
Age	63.5 ± 9.7	65.1 ± 8.6	0.53
Education	15.7 ± 2.5	15.6 ± 3.1	0.86
MMSE	28.0 ± 3.5	28.5 ± 1.6	0.53
CGG repeats	88.1 ± 16.5	83.3 ± 18.1	0.39
Duration in trial (days)	361.8 ± 99.5	350.7 ± 64.3	0.65
FXTAS Stage	3.0 ± 0.9	3.1 ± 0.8	0.75

Abbreviation: MMSE = Mini-Mental State Examination. FXTAS = fragile X-associated tremor/ataxia syndrome.

**Table 2:** Behavioral performance during auditory “oddball” task: Mean ± SD.

	Memantine			Placebo		
	Baseline	1-year	Δ	Baseline	1-year	Δ
RT (ms)	503 ± 90	505 ± 82	2.1 ms	500 ± 79	524 ± 91	24 ms
Accuracy %	95.2 ± 11.7	96.6 ± 7.5	1.4%	96.3 ± 4.7	94.8 ± 9.8	−1.5%
|Count-Hit|	2.52 ± 2.95	1.27 ± 1.79	−1.24	1.43 ± 1.14	2.07 ± 2.39	0.65
*d'*	6.02 ± 1.86	6.72 ± 2.35	0.70	5.79 ± 2.03	5.64 ± 1.97	−0.15

Abbreviations: 1-year* = *1-year follow-up; RT = response time; Δ = 1-year − baseline; *d*′* = *d-prime, the sensitivity measure in signal detection theory, calculated by subtracting the normalized values of the false alarm rates from that of the hit rates.

## References

[b1] Seltzer M. M. *et al.* Prevalence of CGG Expansions of the FMR1 Gene in a US Population-Based Sample. Am J Med Genet B Neuropsychiatr Genet 159B, 589–597, 10.1002/ajmg.b.32065 (2012). 22619118 PMC3391968

[b2] Hagerman R. & Hagerman P. Advances in Clinical and Molecular Understanding of the FMR1 Premutation and Fragile x-Associated Tremor/Ataxia Syndrome. Lancet Neurol 12, 786–798 (2013). 23867198 10.1016/S1474-4422(13)70125-X PMC3922535

[b3] Hagerman P. J. & Hagerman R. J. Fragile X-associated tremor/ataxia syndrome. Ann N Y Acad Sci 1338, 58–70, 10.1111/nyas.12693 (2015). 25622649 PMC4363162

[b4] Grigsby J. *et al.* Cognitive Profile of Fragile X Premutation Carriers with and without Fragile X-Associated Tremor/Ataxia Syndrome. Neuropsychology 22, 48–60, 10.1037/0894-4105.22.1.48 (2008). 18211155

[b5] Brega A. G. *et al.* The Primary Cognitive Deficit among Males with Fragile X-Associated Tremor/Ataxia Syndrome (FXTAS) is a Dysexecutive Syndrome. J Clin Exp Neuropsychol 30, 853–869, 10.1080/13803390701819044 (2008). 18608667 PMC4098148

[b6] Hunter J. E. *et al.* No Evidence for a Difference in Neuropsychological Profile Among Carriers and Noncarriers of the FMR1 Premutation in Adults Under the Age of 50. Am J Hum Genet 83, 692–702, 10.1016/j.ajhg.2008.10.021 (2008). 19026394 PMC2668066

[b7] Hunter J. E., Epstein M. P., Tinker S. W., Abramowitz A. & Sherman S. L. The FMR1 Premutation and Attention-Deficit Hyperactivity Disorder (ADHD): Evidence for a Complex Inheritance. Behav genet 42, 415–422, 10.1007/s10519-011-9520-z (2012). 22101959 PMC3696489

[b8] Farzin F. *et al.* Autism Spectrum Disorders and Attention-Deficit/Hyperactivity Disorder in Boys with the Fragile X Premutation. J Dev Behav Pediatr 27, 137–144 (2006). 10.1097/00004703-200604002-00012 16685180

[b9] Yang J.-C. *et al.* Phenotypes of Hypofrontality in Older Female Fragile X Premutation Carriers. Ann Neurol 74, 275–283 (2013). 23686745 10.1002/ana.23933 PMC3906211

[b10] Cornish K. M., Hocking D. R., Moss S. A. & Kogan C. S. Selective Executive Markers of At Risk Profiles Associated with the Fragile X Premutation. Neurology 77, 618–622 (2011). 21775729 10.1212/WNL.0b013e3182299e59 PMC3159093

[b11] Hashimoto R., Backer K. C., Tassone F., Hagerman R. J. & Rivera S. M. An FMRI Study of the Prefrontal Activity During the Performance of a Working Memory Task in Premutation Carriers of the Fragile X Mental Retardation 1 Gene with and without Fragile X-Associated Tremor/Ataxia Syndrome (FXTAS). J Psychiatr Res 45, 36–43, 10.1016/j.jpsychires.2010.04.030 (2011). 20537351 PMC2978252

[b12] Greco C. M. *et al.* Neuropathology of Fragile X-Associated Tremor/Ataxia Syndrome (FXTAS). Brain 129, 243–255, 10.1093/brain/awh683 (2006). 16332642

[b13] Brunberg J. A. *et al.* Fragile X Premutation Carriers: Characteristic MR Imaging Findings of Adult Male Patients with Progressive Cerebellar and Cognitive Dysfunction. AJNR Am J Neuroradiol 23, 1757–1766 (2002). 12427636 PMC8185834

[b14] Hunsaker M. R., Kim K., Willemsen R. & Berman R. F. CGG Trinucleotide Repeat Length Modulates Neural Plasticity and Spatiotemporal Processing in a Mouse Model of the Fragile X Premutation. Hippocampus 22, 2260–2275, 10.1002/Hipo.22043 (2012). 22707411 PMC3449027

[b15] Cao Z. *et al.* Clustered Burst Firing in FMR1 Premutation Hippocampal Neurons: Amelioration with Allopregnanolone. Hum Mol Genet 21, 2923–2935, 10.1093/hmg/dds118 (2012). 22466801 PMC3373240

[b16] Liu J. *et al.* Signaling Defects in iPSC-Derived Fragile X Premutation Neurons. Hum Mol Genet 21, 3795–3805, 10.1093/hmg/dds207 (2012). 22641815 PMC3412379

[b17] Reisberg B. *et al.* Memantine in Moderate-to-Severe Alzheimer’s Disease. N Engl J Med 348, 1333–1341 (2003). 12672860 10.1056/NEJMoa013128

[b18] Seritan A. L. *et al.* Memantine for Fragile X-Associated Tremor/Ataxia Syndrome: A Randomized, Double-Blind, Placebo-Controlled Trial. J Clin Psychiatry 75, 264–271 (2014). 24345444 10.4088/JCP.13m08546 PMC4296896

[b19] Yang J. C. *et al.* Memantine Effects on Verbal Memory in Fragile X-associated Tremor/Ataxia Syndrome (FXTAS): a Double-Blind Brain Potential Study. Neuropsychopharmacology 39, 2760–2768, 10.1038/npp.2014.122 (2014). 24871547 PMC4200486

[b20] Luck S. J., Ford J. M., Sarter M. & Lustig C. CNTRICS Final Biomarker Selection: Control of Attention. Schinzophr Bull 38, 53–61 (2012). 10.1093/schbul/sbr065 PMC3245597 21765166

[b21] Leiser S. C., Dunlop J., Bowlby M. R. & Devilbiss D. M. Aligning Strategies for Using EEG as a Surrogate Biomarker: A Review of Preclinical and Clinical Research. Biochem Pharmacol 81, 1408–1421 (2011). 20937262 10.1016/j.bcp.2010.10.002

[b22] Polich J. Neuropsychology of P300. (Oxford University Press, 2012).

[b23] Herrmann C. S. & Knight R. T. Mechanisms of Human Attention: Event Related Potentials and Oscillations. Neurosci Biobehav Rev 25, 465–476 (2001). 11595268 10.1016/s0149-7634(01)00027-6

[b24] Yang J.-C. *et al.* Neural Substrates of Executive Dysfunction in Fragile X-Associated Tremor/Ataxia Syndrome (FXTAS): A Brain Potential Study. Cereb Cortex 23, 2657–2666 (2013). 22918986 10.1093/cercor/bhs251 PMC3792740

[b25] Tikhonravov D. *et al.* Dose-Related Effects on Memantine on a Mismatch Negativity-Like Response in Anesthetized Rats. Neuroscience 167, 1175–1182 (2010). 20298759 10.1016/j.neuroscience.2010.03.014

[b26] Connolly P. M. *et al.* The Effects of Ketamine Vary Among Inbred Mouse Strains and Mimic Schizophrenia for the P80, but Not P20 or N40 Auditory ERP Components. Neurochem 29, 1179–1188 (2004). 10.1023/b:nere.0000023605.68408.fb 15176475

[b27] Siegel S. J. *et al.* Effects of strain, novelty, and NMDA blockade on auditory-evoked potentials in mice. Neuropsychopharmacology 28, 675–682, 10.1038/sj.npp.1300087 (2003). 12655312

[b28] Ehlers C. L., Kaneko W. M., Chaplin W. & Chaplin R. I. Effects of Dizocilpine (MK-801) and Ethanol on the EEG and Event-Related Potentials (ERPS) in Rats. Neuropharmacology 31, 369–378 (1992). 1522954 10.1016/0028-3908(92)90069-2

[b29] Ehrlichman R. S. *et al.* N-methyl-d-aspartic acid receptor antagonist-induced frequency oscillations in mice recreate pattern of electrophysiological deficits in schizophrenia. Neuroscience 158, 705–712, 10.1016/j.neuroscience.2008.10.031 (2009). 19015010

[b30] Korostenskaja M., Nikulin V. V., Kicic D., Nikulina A. V. & Kahkonen S. Effects of NMDA Receptor Antagonist on Mismatch Negativity. Brain Res Bull 72, 275–283 (2007). 17452287 10.1016/j.brainresbull.2007.01.007

[b31] Umbricht D. *et al.* Midlatency auditory event-related potentials in mice: comparison to midlatency auditory ERPs in humans. Brain Res 1019, 189–200, 10.1016/j.brainres.2004.05.097 (2004). 15306253

[b32] Umbricht D., Koller R., Vollenweider F. X. & Schieder L. Mismatch Negativity Predicts Psychotic Experiences Induced by NMDA Receptor Antagonist in Healthy Volunteers. Biol Psychiatry 51, 400–406 (2002). 11904134 10.1016/s0006-3223(01)01242-2

[b33] McFall R. M. & Treat T. A. Quantifying the information value of clinical assessments with signal detection theory. Annu Rev Psychol 50, 215–241 (1999). 15012460 10.1146/annurev.psych.50.1.215

[b34] Thal L. J. *et al.* The role of biomarkers in clinical trials for Alzheimer disease. Alzheimer Dis Assoc Disord 20, 6–15, 10.1097/01.wad.0000191420.61260.a8 (2006). 16493230 PMC1820855

[b35] Gazzaniga M. S., Ivry R. B. & Mangun G. R. Cognitive Neuroscience: the biology of the mind. 3 edn, (W.W. Norton, 2008).

[b36] Crowley K. E. & Colrain I. M. A Review of the Evidence for P2 being an Independent Component Process: Age, Sleep, and Modality. Clin Neurophysiol 115, 732–744 (2004). 15003751 10.1016/j.clinph.2003.11.021

[b37] Melara R. D., Tong Y. & Rao A. Control of Working Memory: Effects of Attention Training on Target Recognition and Distractor Salience in an Auditory Selection Task. Brain Res 68–67 (2012). 22099165 10.1016/j.brainres.2011.10.036

[b38] Verleger R., Heide W., Butt C. & Kompf D. Reduction of P3b in patients with temporo-parietal lesions. Brain Res Cogn Brain Res 2, 103–116 (1994). 7833690 10.1016/0926-6410(94)90007-8

[b39] Amenedo E. & Diaz F. Aging-related changes in processing of non-target and target stimuli during an auditory oddball task. Biol Psychol 48, 235–267 (1998). 9788763 10.1016/s0301-0511(98)00040-4

[b40] Melara R. D., Chen S. & Wang H. Inhibiting change: effects of memory on auditory selective attention. Brain Res Cogn Brain Res 25, 431–442, 10.1016/j.cogbrainres.2005.07.002 (2005). 16157478

[b41] Melara R. D., Rao A. & Tong Y. The Duality of Selection: Excitatory and Inhibitory Processes in Auditory Selective Attention. J Exp Psychol Hum Percept Perform 28, 279–306 (2002). 11999855

[b42] McCabe D. P., Roediger H. L., McDaniel M. A., Balota D. A. & Hambrick D. Z. The Relationship Between Working Memory Capacity and Executive Functioning: Evidence for a Common Executive Attention Construct. Neuropsychology 24, 222–243, 10.1037/A0017619 (2010). 20230116 PMC2852635

[b43] Miyake A. *et al.* The unity and diversity of executive functions and their contributions to complex “frontal lobe” tasks: A latent variable analysis. Cognitive Psychol 41, 49–100, 10.1006/cogp.1999.0734 (2000). 10945922

[b44] Polich J. EEG and ERP assessment of normal aging. Electroencephalogr Clin Neurophysiol 104, 244–256 (1997). 9186239 10.1016/s0168-5597(97)96139-6

[b45] Ponton C. W., Eggermont J. J., Kwong B. & Don M. Maturation of human central auditory system activity: evidence from multi-channel evoked potentials. Clin Neurophysiol 111, 220–236 (2000). 10680557 10.1016/s1388-2457(99)00236-9

[b46] Knight R. T., Scabini D., Woods D. L. & Clayworth C. The effects of lesions of superior temporal gyrus and inferior parietal lobe on temporal and vertex components of the human AEP. Electroencephalogr Clin Neurophysiol 70, 499–509 (1988). 2461284 10.1016/0013-4694(88)90148-4

[b47] Wang J. Y., Hagerman R. J. & Rivera S. M. A multimodal imaging analysis of subcortical gray matter in fragile X premutation carriers. Mov disord 28, 1278–1284 (2013). 23649693 10.1002/mds.25473 PMC3785985

[b48] Moore C. J. *et al.* The effect of pre-mutation of X chromosome CGG trinucleotide repeats on brain anatomy. Brain 127, 2672–2681 (2004). 15483045 10.1093/brain/awh256

[b49] Broberg B. V. *et al.* Assessment of Auditory Sensory Processing in a Neurodevelopmental Animal Model of Schizophrenia-Gating of Auditory-Evoked Potentials and Prepulse Inhibition. Behav Brain Res 213, 142–147 (2010). 20417666 10.1016/j.bbr.2010.04.026

[b50] de Bruin N. M., Ellenbroek B. A., Cools A. R., Coenen A. M. & van Luijtelaar E. L. Differential effects of ketamine on gating of auditory evoked potentials and prepulse inhibition in rats. Psychopharmacology (Berl) 142, 9–17 (1999). 10102777 10.1007/s002130050856

[b51] Bickel S., Lipp H.-P. & Umbricht D. Early Auditory Sensory Processing Deficits in Mouse Mutants with Reduced NMDA Receptor Function. Neuropharmacology 33, 1680–1690 (2008). 10.1038/sj.npp.1301536 17712349

[b52] Ortigas M. C. *et al.* Improving Fragile X-Associated Tremor/Ataxia Syndrome Symptoms with Memantine and Venlafaxine. J Clin Psychopharmacol 30, 642–644, 10.1097/JCP.0b013e3181f1d10a (2010). 20841969 PMC4022473

[b53] Seeman P., Caruso C. & Lasaga M. Memantine agonist action at dopamine D2^High^ receptors. Synapse 62, 149–53, (2008). 18000814 10.1002/syn.20472

[b54] Niu Y. Q. *et al.* Parkinsonism in fragile X-associated tremor/ataxia syndrome (FXTAS): revisited. Parkinsonism Relat Disord 20, 456–459, 10.1016/j.parkreldis.2014.01.006 (2014). 24491663 PMC4019503

[b55] Kähkönen S. *et al.* Dopamine modulates involuntary attention shifting and reorienting: an electromagnetic study. Clin Neurophysiol 113, 1894–1902 (2002). 12464326 10.1016/s1388-2457(02)00305-x

[b56] Sani G. *et al.* The Role of Memantine in the Treatment of Psychiatric Disorders Other Than the Dementias: a review of current preclinical and clinical evidence. CNS Drugs 26, 663–690, 10.2165/11634390-000000000-00000 (2012). 22784018

[b57] Serra G. *et al.* Memantine: New prospective in bipolar disorder treatment. World J Psychiatry 4, 80–90, 10.5498/wjp.v4.i4.80 (2014). 25540723 PMC4274590

[b58] Olichney J. M. *et al.* Abnormal N400 word repetition effects in fragile X-associated tremor/ataxia syndrome. Brain 133, 1438–1450, 10.1093/brain/awq077 (2010). 20410144 PMC2859155

